# Supplementary material for: Histone modification cross talk between a host and pathogen
Source: Proc Natl Acad Sci U S A. 2026 Aug 24;123(35):e2611585123. doi: 10.1073/pnas.2611585123 (PMC13534545; doi:10.1073/pnas.2611585123)
Supplement: Supplementary file 1 [file pnas.2611585123.sapp.pdf]

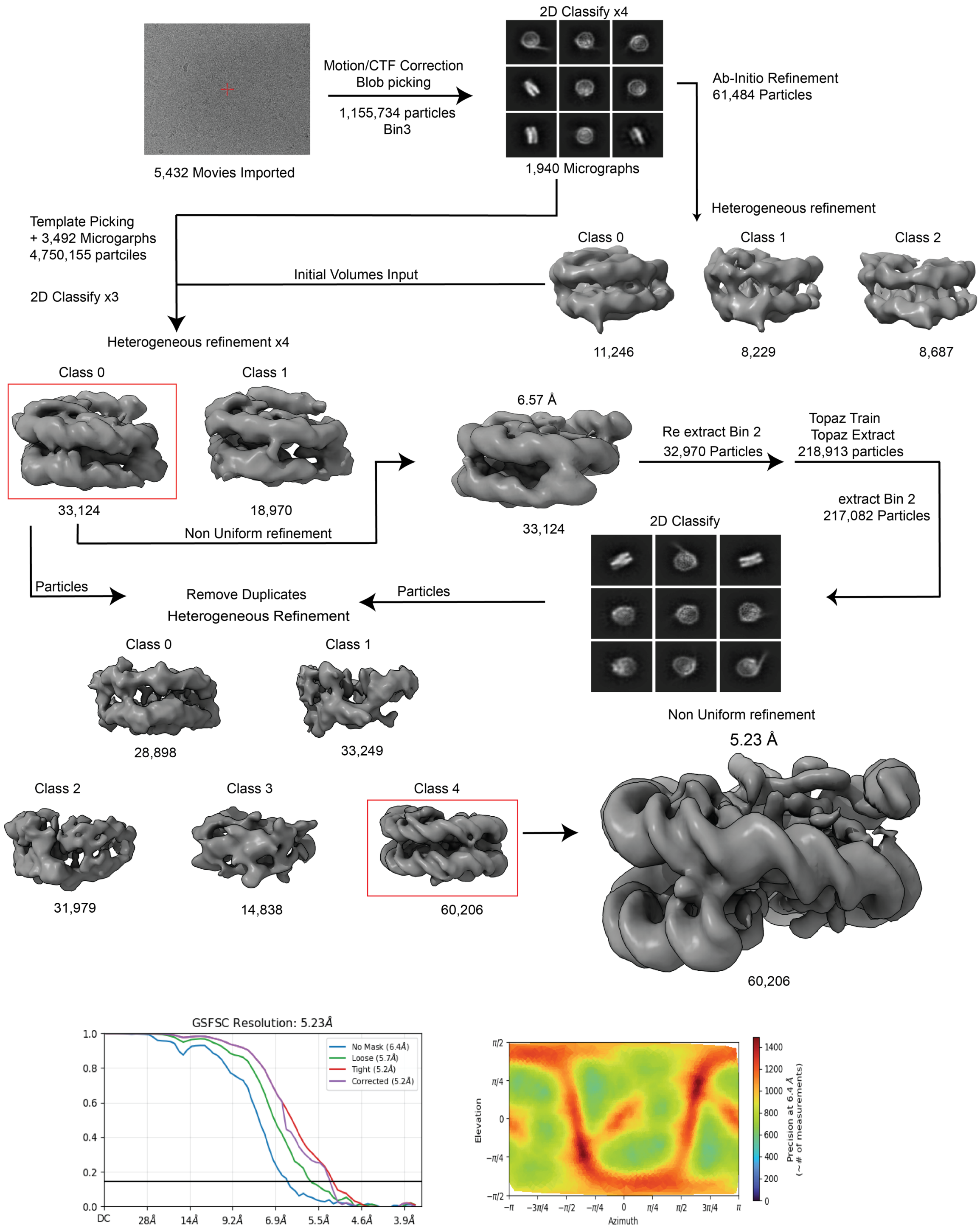

Figure S1: Cryo-EM processing pipeline for the H4K12me1 nucleosome

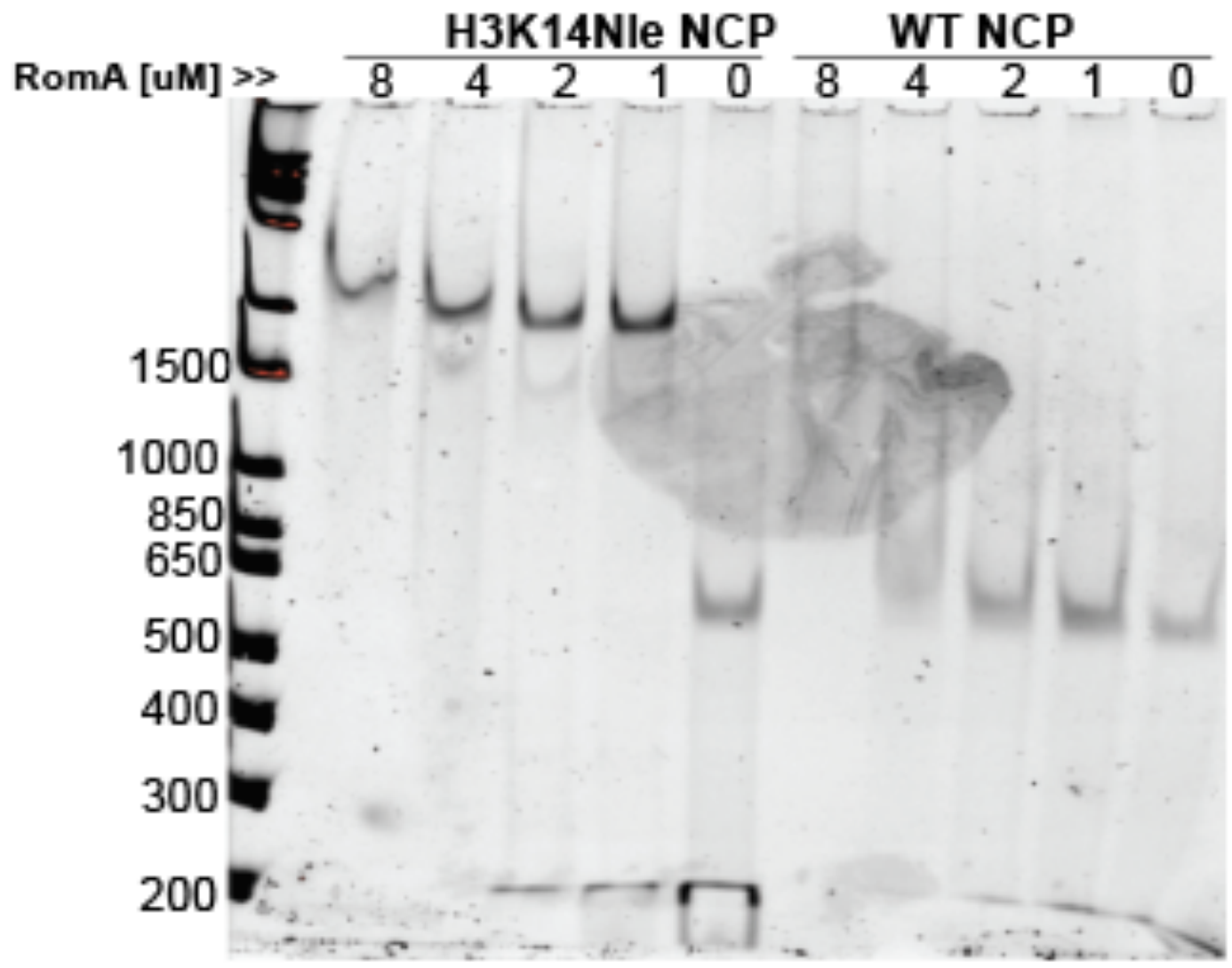

Figure S2: H3K14Nle NCP purification and crosslinking

7% Native Page showing RomA bound to Wildtype Nucleosome vs H3K14Nle Nucleosomes in the presence of SAM. RomA = 0-8uM, SAM = 200uM, Nucleosome = 200nM

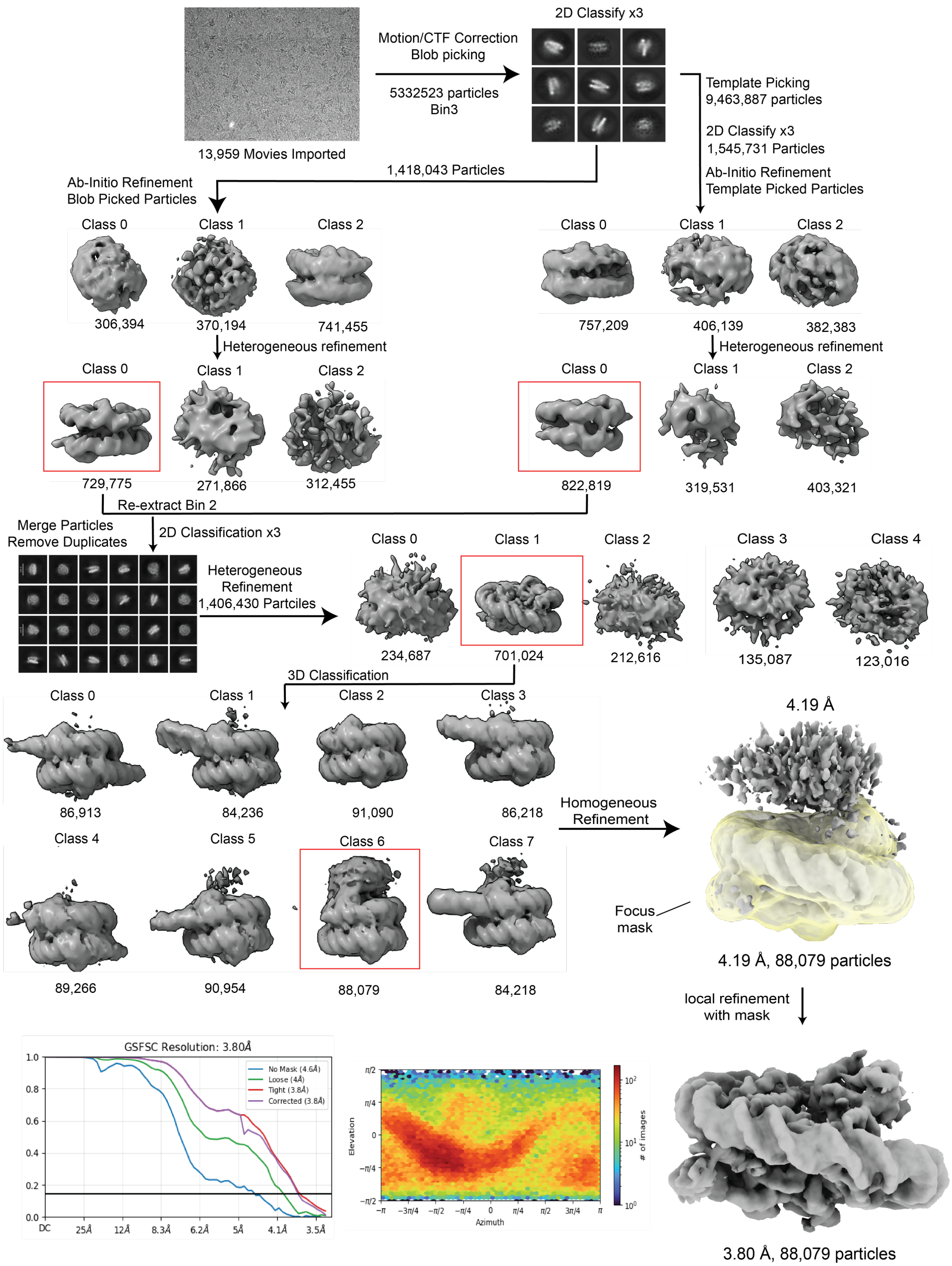

Figure S3: Cryo-EM processing pipeline for the RomA H3K14Nle nucleosome complex

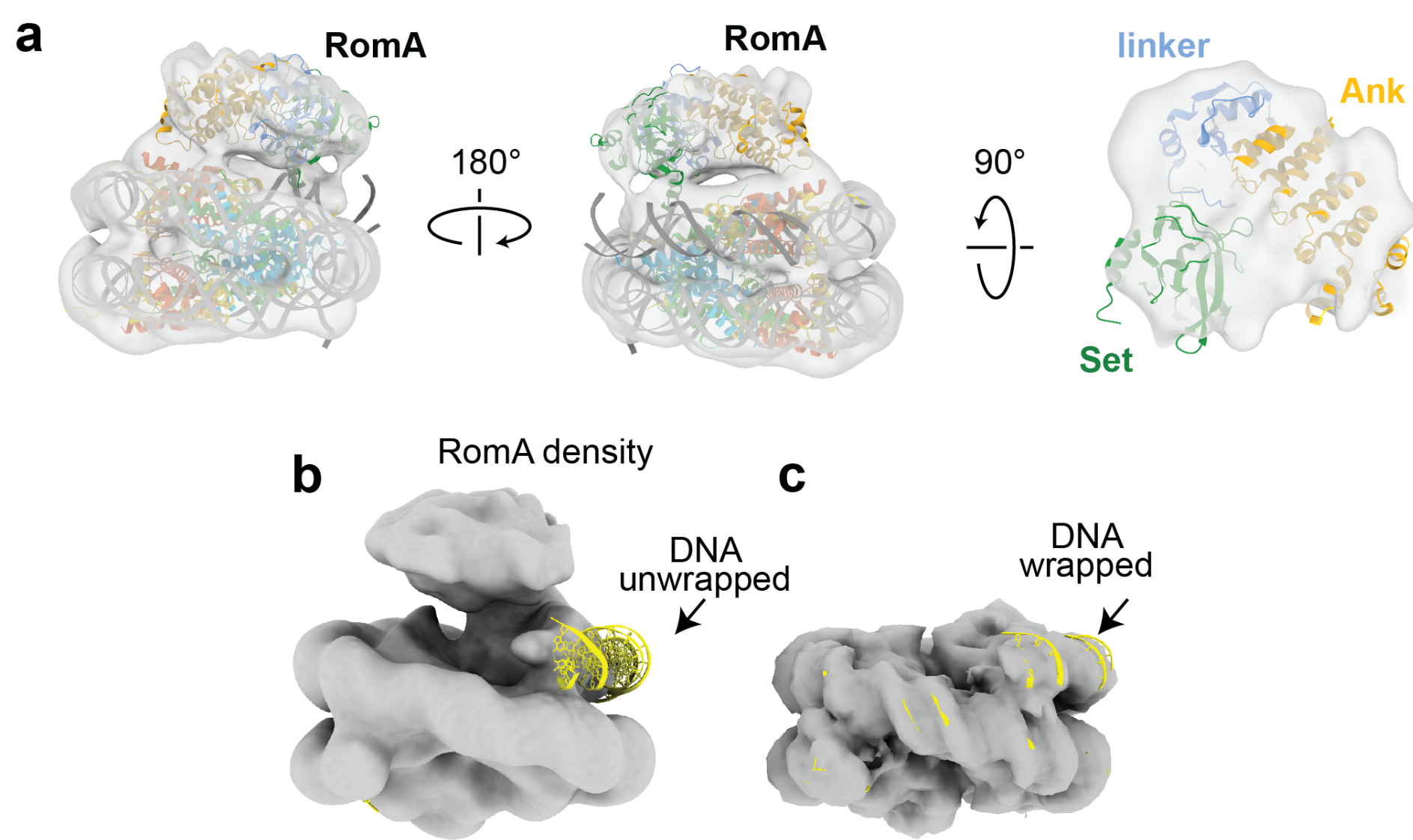

Figure S4: Structural details of the RomA-nucleosome complex

a) Cryo-EM structure of the RomA-([H3K14Nle]2) complex filtered to 15Å for clarity. The Cryo-EM density is shown as a semi-transparent gray surface and the structures of a nucleosome (PDB: 8YBJ) and LegAS4 (PDB: 5CZY) are docked into the Cryo-EM density. b) RomA-([H3K14Nle]2) complex filtered to 15Å showing that density for DNA is missing on the side of the nucleosome where RomA is observed. c) Cryo-EM density for the fully wrapped nucleosome (3D classification, Class 2, Fig. S3) where no RomA density is observed. DNA from the nucleosome (PDB: 8YBJ) is superimposed with the cryo-EM maps and colored yellow.

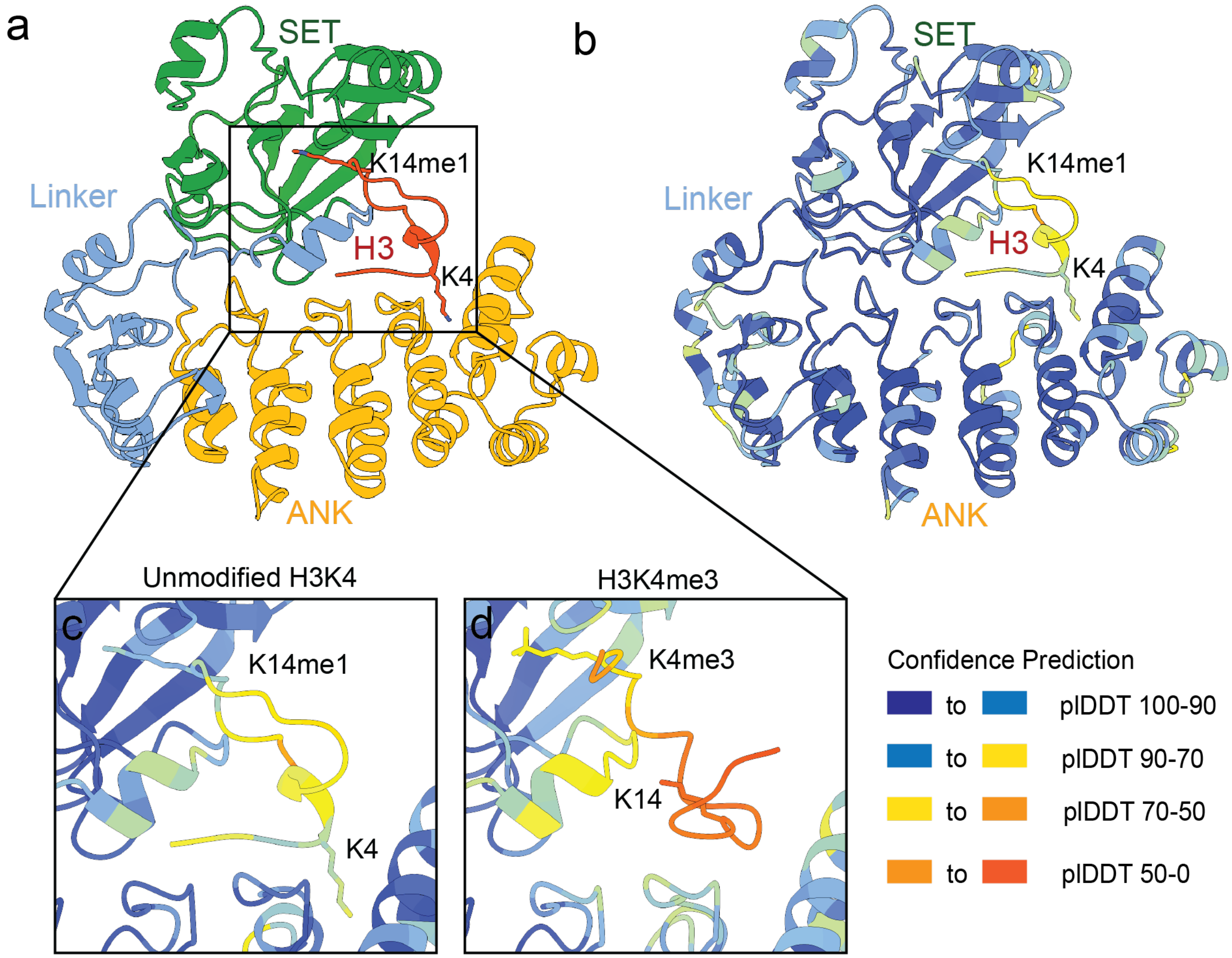

Figure S5: AlphaFold prediction of RomA bound to an H3 peptides

a) Alphafold prediction of RomA bound to a H3K14me1 (1-20) peptide colored by domains. H3K4 and H3K14me1 are depicted as sticks. b) Alphafold prediction as in a but colored by PIDDT scores. c) Close up view of the H3K14me1 peptide. d) Close up view of the H3K4me3 peptide showing loss of interactions with the Ank domain.
